# A novel compound heterozygous *BEST1* gene mutation in two siblings causing autosomal recessive bestrophinopathy

**DOI:** 10.1186/s12886-022-02703-5

**Published:** 2022-12-16

**Authors:** Obaid Imtiyazul Haque, Anbukayalvizhi Chandrasekaran, Faisal Nabi, Owais Ahmad, João Pedro Marques, Tanweer Ahmad

**Affiliations:** 1 Massachusetts General Hospital, Harvard Medical School, Boston, USA; 2 MedGenome Labs Limited, Bengaluru, India; 3 Interdisciplinary Biotechnology Unit, Aligarh Muslim University, Aligarh, India; 4 Department of Microbiology and Cell Biology, Indian Institute of Science, Bangalore, India; 5 Ophthalmology Unit, Centro Hospitalar E Universitário de Coimbra (CHUC), Coimbra, Portugal; 6 Northampton General Hospital, Northampton, UK

**Keywords:** Autosomal recessive bestrophinopathy, BEST1, Bestrophin-1, Inherited retinal dystrophy, Genetics

## Abstract

**Purpose:**

To describe the clinical features, imaging characteristics, and genetic test results associated with a novel compound heterozygous mutation of the *BEST1* gene in two siblings with autosomal recessive bestrophinopathy.

**Methods:**

Two siblings underwent a complete ophthalmic examination, including dilated fundus examination, fundus photography, fundus autofluorescence imaging, spectral-domain optical coherence tomography, fluorescein angiography, electroretinography, and electrooculography. A clinical diagnosis of autosomal recessive bestrophinopathy was established based on ocular examination and multimodal retinal imaging. Subsequently, clinical exome sequencing consisting of a panel of 6670 genes was carried out to confirm the diagnosis and assess genetic alterations in the protein-coding region of the genome of the patients. The identified mutations were tested in the two affected siblings and one of their parents.

**Results:**

Two siblings (a 17-year-old female and a 15-year-old male) presented with reduced visual acuity and bilaterally symmetrical subretinal deposits of hyperautofluorescent materials in the posterior pole, which showed staining in the late phase of fluorescein angiogram. Spectral-domain optical coherence tomography demonstrated hyperreflective subretinal deposits and subretinal fluid accumulation. Both patients shared two mutations in the protein-coding region of the *BEST1* gene, c.103G > A, p.(Glu35Lys) and c.313C > A, p.(Arg105Ser) (a novel disease-causing mutation). Sanger sequencing confirmed that the unaffected mother of the proband was carrying p.(Glu35Lys) variant in a heterozygous state.

**Conclusions:**

We have identified and described the phenotype of a novel disease-causing mutation NM_004183.4:c.313C > A, p.(Arg105Ser) in a heterozygous state along with a previously reported mutation NM_004183.4:c.103G > A, p.(Glu35Lys) of the *BEST1* gene in two related patients with autosomal recessive bestrophinopathy.

**Supplementary Information:**

The online version contains supplementary material available at 10.1186/s12886-022-02703-5.

## Introduction

The *BEST1* (alternatively *VMD2, RP50, BMD*) gene located on chromosome GRCh38 11q12.3 encodes a transmembrane pentameric protein consisting of 585 amino acids with a highly conserved N-terminal region followed by four transmembrane domains (amino acids 1–390) and a carboxy-terminal region (amino acids 391–585) [1]. Structural models of the *BEST1* propose the N- and C-termini as being cytosolic with four transmembrane domains (domains 1, 2, 5, and 6) and two cytoplasmic domains (domains 3 and 4) [2, 3]. The protein is predominantly expressed in the basolateral plasma membrane of the retinal pigment epithelium (RPE) and functions as a calcium-activated chloride channel (CaCC) which regulates the flow of chloride and other monovalent anions across cellular membranes in response to intracellular calcium levels [4–8]. Mutation of the *BEST1* gene has been associated with lipofuscin accumulating within and beneath the RPE and degeneration of the RPE and the overlying photoreceptors [9]. A wide range of ocular phenotypes resulting from mutations in the *BEST1* gene have been described and are collectively termed *bestrophinopathies*. [10, 11] Autosomal recessive bestrophinopathy (ARB) may result from a total absence (null phenotype) of functional *BEST1* protein in the RPE, [12, 13] improper localization to the cell membrane with intact anion channel activity, [14] or lack of the anion channel activity [15].

Schatz et al., in 2006, first described a variant of Best macular dystrophy in two members of a Swedish family presenting with reduced vision, multifocal retinal deposits, and intraretinal cystic changes, harboring biallelic mutations in the *BEST1* gene. [16] In 2008, Burgess et al. coined the term *autosomal recessive bestrophinopathy* (ARB) and identified it as the third distinct phenotype resulting from mutations in the *BEST1* gene. [12] Other described phenotypes associated with pathogenic variants of the *BEST1* gene include Best vitelliform macular dystrophy (BVMD) [17, 18], adult vitelliform macular dystrophy (AVMD), autosomal dominant vitreoretinochoroidopathy (ADVIRC), [19, 20] autosomal dominant microcornea, rod-cone dystrophy, early-onset cataract, and posterior staphyloma (MRCS) [11], rod-cone dystrophy and retinitis pigmentosa [11, 21]. In contrast to other phenotypes of bestrophinopathies that result from dominant mutations, ARB is associated with recessive biallelic mutations in the *BEST1* gene [12, 22, 23].

Patients with ARB typically present in the first two decades of life but may remain asymptomatic as late as the fifth decade [12, 15, 24]. The clinical features of ARB include a gradual and progressive visual loss, hyperopia, predominantly peri-macular sub-retinal yellowish deposits of lipofuscin, seen as hyperautofluorescent areas, accumulation of subretinal and/or intraretinal fluid, absence of light peak in electrooculography, normal or reduced electroretinogram, and sometimes associated with shallow anterior chambers and reduced axial length predisposing the affected patients to angle-closure glaucoma. [12, 25, 26] Full-field electroretinography is typically normal early on in the disease and shows abnormal results from late childhood or adolescence, indicating generalized rod and cone dysfunction. In addition, pattern electroretinography evidence of macular dysfunction is also seen. [12] This article presents the results of clinical evaluation, multimodal imaging, electrophysiological tests, and genetic investigations of two siblings with ARB.

## Methods

### Clinical investigation

Clinical investigations in patients included a detailed history and physical examination, slit-lamp biomicroscopy, indirect ophthalmoscopy, fundus photography, fundus autofluorescence imaging (FAF), optical coherence tomography (OCT), fluorescein angiography (FA), full-field electroretinography (ERG), and electrooculography (EOG). The ERG and EOG were performed per the guidelines of the International Society for Clinical Electrophysiology of Vision (www.iscev.org).

### Genetic analysis

#### Whole exome sequencing

##### DNA isolation, exome library preparation, and sequencing

DNA was isolated from the patient’s whole blood sample using QIAamp DNA Blood Mini Kit (QIAGEN, CA, US) and subjected to targeted gene capture using MedGenome Clinical Exome (Ver. 4) which captures a panel of 6670 protein-coding genes. The libraries thus generated were sequenced to mean coverage of > 80-100X on the Illumina HiSeq 4000 sequencing platform (Illumina, CA, US). 100% of the protein-coding region of the *BEST1* gene was covered.

##### Variant calling and annotation

The Genome Analysis Toolkit (GATK) best practices framework was followed to identify the variants in the sample using Sentieon (v201808.07)**.** The sequencing reads were aligned to the human reference genome (GRCh38.p13) using the Sentieon aligner. Sentieon’s version of GATK (IndelRealigner) was used to perform local realignment in regions containing potential indels. Sentieon’s version of GATK Toolkit – BaseRecalibrator was used to recalibrate the quality scores of all the reads [27]. Sentieon DNASeq (v201808.07) HaplotypeCaller was used to identify variants. Gene annotation of the variants was performed using the VEP program against the Ensembl release 99 human gene model [28, 29]. In addition to SNVs and small Indels, copy number variants (CNVs) were detected from targeted sequence data using the ExomeDepth (v1.1.10) method [30]. Clinically relevant mutations were annotated using published variants in literature and a set of disease databases—ClinVar [31], OMIM [32] (updated on 11th May 2020), GWAS [33], HGMD (v2020.2) [34], and SwissVar [35].

##### Variant filtering and analysis

To identify candidate variants, we selected the variations if their minor allele frequencies are less than 0.05 in 1000 Genome Project [36], gnomAD [37], dbSNP [38], Exome Variant Server [39], 1000 Japanese Genome [40], and internal Indian population database. The identified variations were classified into pathogenic, likely pathogenic, VUS, likely benign, and benign groups according to the variant interpretation guidelines of the American College of Medical Genetics and Genomics (ACMG) [41]. Furthermore, all nucleotide variants present in *BEST1* were reviewed. The genes and corresponding variants that qualified these filtering criteria were investigated to determine their significance and relevance in Bestrophinopathy. 8.65 Gb of raw sequencing data was generated, of which > 88% of raw reads passed the on-target alignment. > 90% of the targeted base qualified the Phred score Q30.

#### Sanger sequencing

Sanger sequencing was performed in the proband and the mother to validate the variants identified by whole exome sequencing and identify the mutation in the parent. Sanger sequencing was performed with these primers:

F1: 5′-ATCGGTGTCCCTCTCTACCA-3′, R1: 5′-CTATGTGGGCCTATGAGTCTG-3′; F2: 5′-CGTCCTGCCGTTAGCAATG-3′, R2: 5′-CACCTTCAGACACCCGACT-3′. The reference sequence NM_004183 of *BEST1* was used.

### Bioinformatics analysis

The potential functional impact of all the candidate variants was investigated using three programs, including PolyPhen2 (http://genetics.bwh.harvard.edu/pph/, in the public domain), Mutation Taster (http://www.mutationtaster.org/, in the public domain), and SIFT (http://sift.jcvi.org/, in the public domain).

## Results

### Clinical findings

**Fig. 1:**
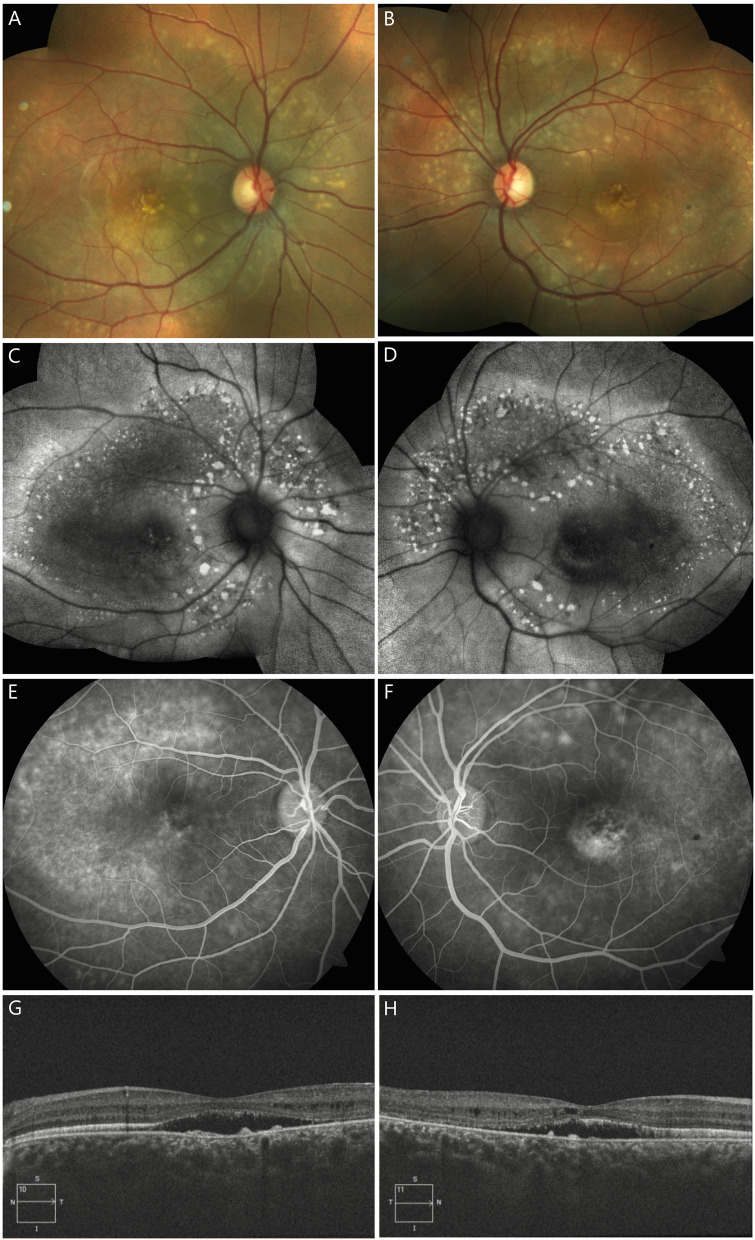
Clinical features of patient A (proband). A 17-year-old otherwise healthy female presented with blurred distance vision in both eyes, which she first noticed when she was 14 years old. Colour fundus photographs (**A**, **B**) show bilateral and symmetrical, multifocal subretinal yellowish deposits in the posterior pole and upper nasal region, with notable peripapillary sparing. The yellowish deposits are hyperautofluorescent on blue light fundus autofluorescence (**C**, **D**) and circumscribe areas of hypoautofluorescence. On fluorescein angiography (E, F), the yellowish deposits show diffuse staining in the late phase. Horizontal spectral-domain optical coherence tomography images through the right and left fovea (**G**, **H**) show center-involving subretinal fluid and thickening of the ellipsoid zone, with elongation of the photoreceptor outer segments and deposits in the subretinal space. Additionally, intraretinal hyporreflective areas predominantly located in the outer nuclear layer can be seen

Patient A (proband), a 17-year-old female, reported blurred distance vision in both eyes for three years. Her best-corrected visual acuity was 6/9 in both eyes, which did not change during the follow-up of 1 year. Slit-lamp examination of the anterior segment of both patients was unremarkable. The axial length measured by optical biometry was 21.80 mm and 21.64 mm in the right and the left eye, respectively. Dilated fundus examination revealed bilateral and symmetrical, multifocal subretinal yellowish deposits in the posterior pole and upper nasal region, with peripapillary sparing (Fig. 1A, B). On FAF, the yellowish deposits appeared as hyper-autofluorescent spots surrounding an area of hypo-autofluorescence (Fig. 1C, D). The deposits showed staining in the late phase of the fluorescein angiogram (Fig. 1E, F). On OCT, subretinal fluid and intraretinal hyporeflective spaces (schisis) located predominantly in the outer nuclear layer (ONL) were seen along with elongated photoreceptors and hyperreflective deposits in the subretinal space bilaterally (Fig. 1G, H). The electroretinogram (ERG) was normal, and an absent light peak was noted on EOG.

**Fig. 2:**
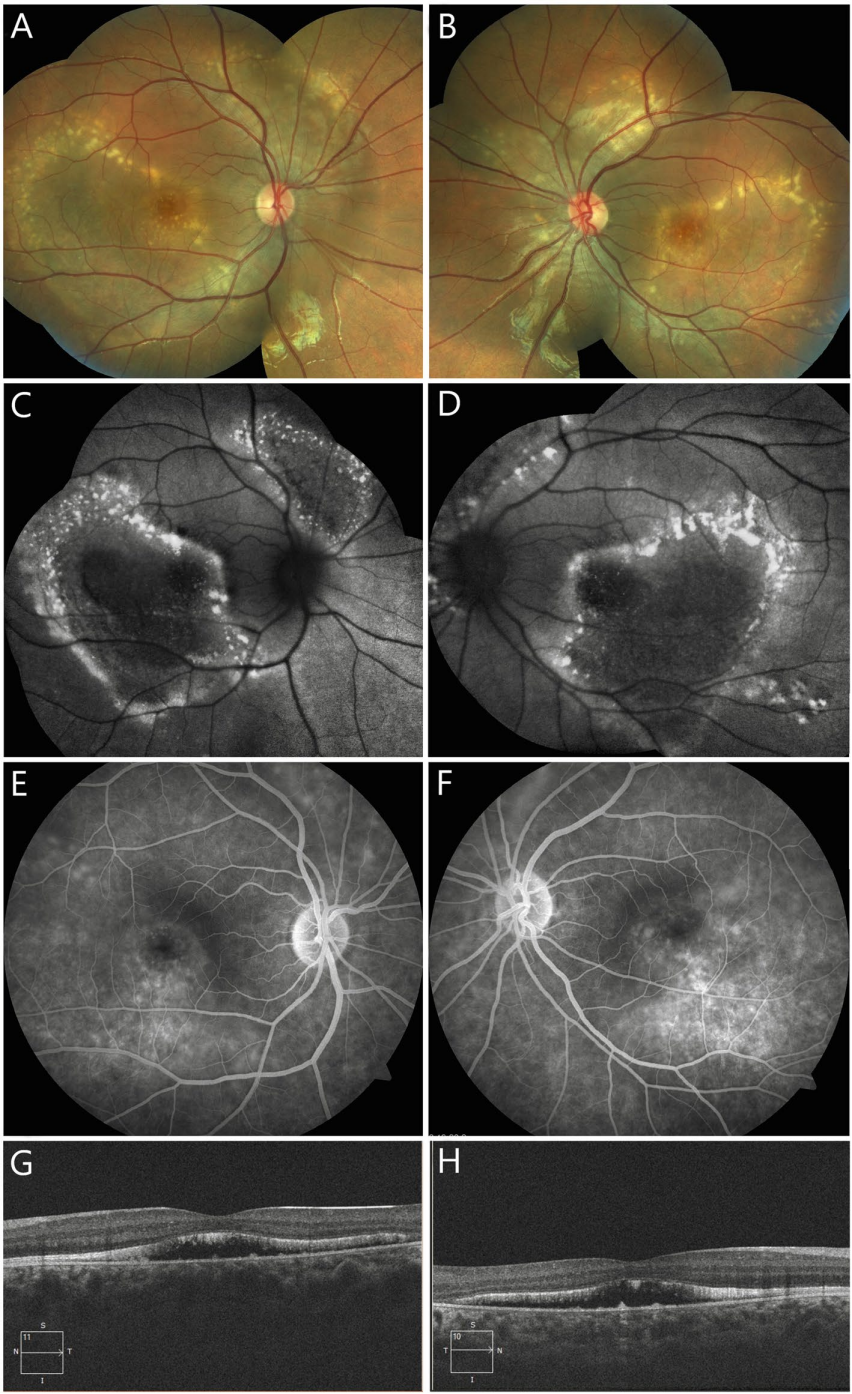
Clinical features of patient B. A 15-year-old otherwise healthy male presented with a unilateral decrease in visual acuity and inward deviation of the left eye, which was first noticed by his parents when he was four. On colour fundus photographs of the right and the left eye (**A**, **B**), two separate areas of bilateral and symmetrical, multifocal subretinal yellowish deposits are seen in the posterior pole and upper nasal region, with notable peripapillary sparing. On blue light fundus autofluorescence (**C**, **D**), the yellowish deposits are hyperautofluorescent, and circumscribed areas show hypoautofluorescence. In the late phase of fluorescein angiography (**E**, **F**), diffuse staining, seen as diffuse hyperfluorescence of the yellowish deposits is observed. Horizontal spectral-domain optical coherence tomography scans through the fovea (**G**, **H**) show center-involving subretinal fluid and thickening of the ellipsoid zone, with elongation of the photoreceptor outer segments and deposits in the subretinal area

Patient B, a 15-year-old male, reported a unilateral decrease in visual acuity and inward deviation of the left eye since the age of four. On ocular examination, he was found to have left esotropia of 15 prism diopters with prescribed correction and 25 prism diopters without the correction for distance. His best-corrected visual acuity was 6/6 in his right eye and 5/60 in his left eye, with an accommodative-convergence over accommodation (AC/A) ratio of 2:1. Slit-lamp examination of the anterior segment was unremarkable. The axial length measured by optical biometry was 21.61 mm and 21.54 mm in the right and the left eye, respectively. Dilated fundus examination revealed two circumscribed areas of bilaterally symmetrical, multifocal subretinal yellowish deposits, one in the posterior pole and the other in the upper nasal region, with peripapillary sparing. (Fig. 2A, B). The yellowish lesions were hyper-autofluorescent surrounding an area of hypo-autofluorescence on FAF (Fig. 2C, D) and showed staining in the late phase of fluorescein angiogram (Fig. 2E, F). On OCT, subretinal fluid (seen as subfoveal hyporeflective space), elongated photoreceptors along with hyperreflective deposits in the subretinal area were observed bilaterally (Fig. 2G, H). The electroretinogram (ERG) was normal, and an absent light peak was noted on EOG.

**Fig. 3:**
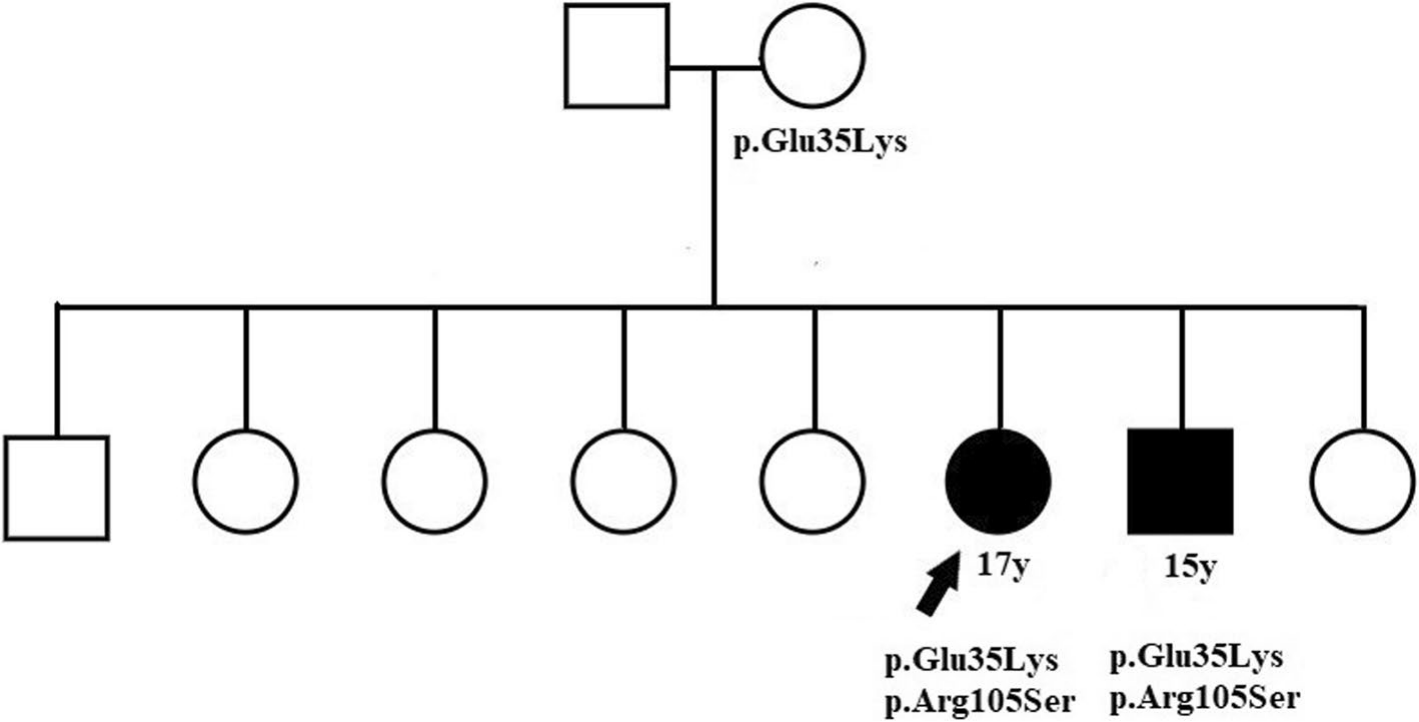
Pedigree of the family with two affected members. Patient A, a 17-year-old female (black arrow), and Patient B, a 15-year-old male

The patients were born of a non-consanguineous marriage from parents of North Indian descent. The parents and unaffected siblings were examined; however, no ocular or systemic abnormalities were observed. (Fig. 3). Both affected siblings were treated with topical carbonic anhydrase inhibitors and followed up for one year. The subfoveal fluid did not improve after one year of treatment. The clinical, imaging, and electrophysiological findings of the affected patients are summarized in Table 1.

**Table 1:** Clinical profile of the patients

Patient	Age/sex	Axial length/ AC depth (mm)		BCVA, spherical equivalents		FAF	OCT	ERG	EOG
		OD	OS	OD	OS				
A	17/F	21.80/2.65	21.64/ 2.71	6/9 (-1.25)	6/9 (-1.50)	Hyper autoflourescent deposits	Intra-retinal spaces (schisis), sub-retinal deposits, subretinal fluid	Normal	Absent light peak
B	15/M	21.61/ 3.48	21.54/ 3.53	6/6 (+ 5.00)	5/60 (+ 5.00)	Hyper autoflourescenct deposits	Sub-retinal fluid and deposit	Normal	Absent light peak

*AC* Anterior Chamber, *BCVA* Best-corrected visual acuity, *FAF* Fundus autofluorescence, *OCT* Optical Coherence Tomography, *ERG* electroretinogram, *EOG* Electrooculogram

### Genetic findings

**Fig. 4:**
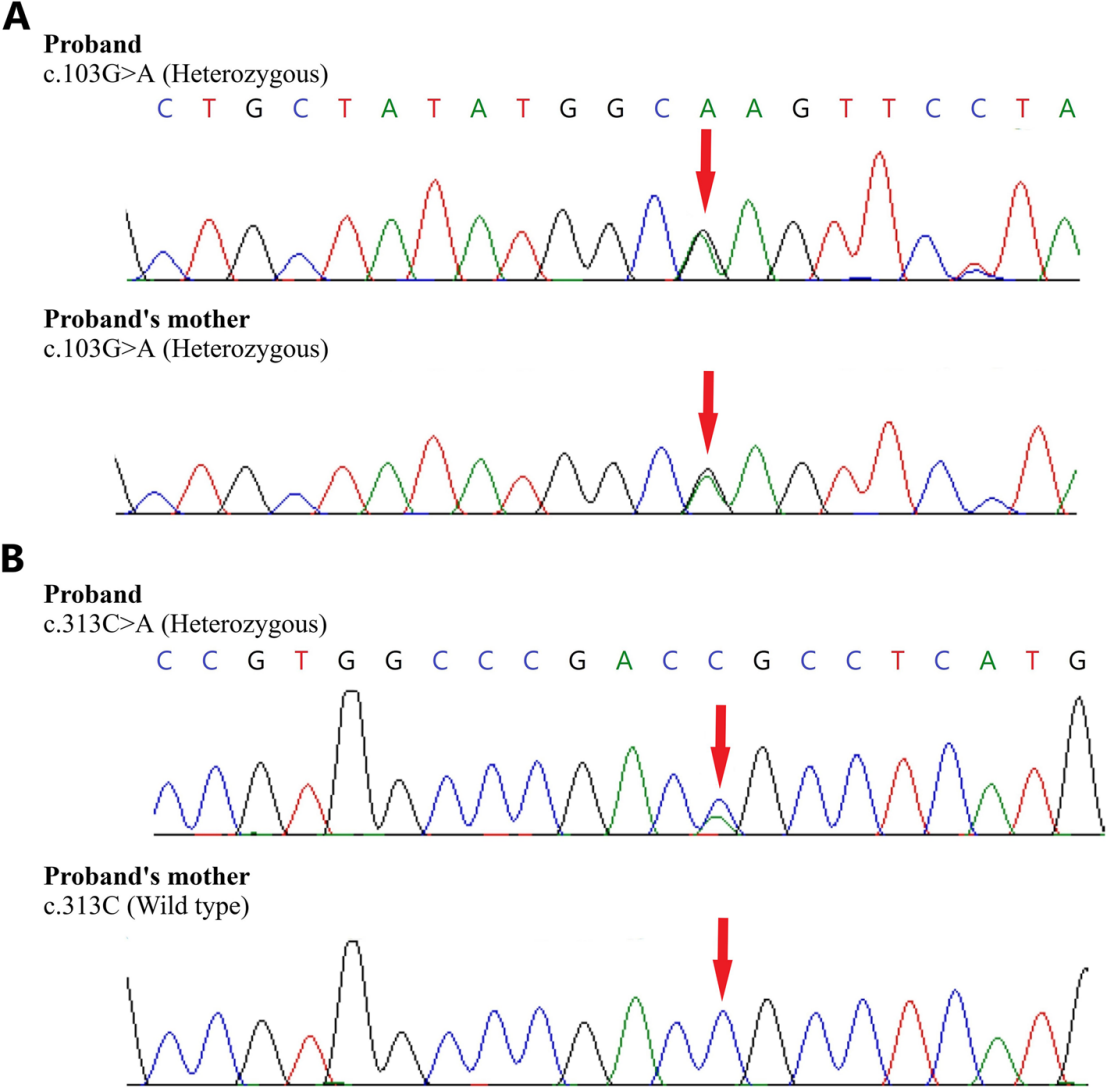
**A** Sequence chromatogram and alignment to the reference sequence showing the variant in exon 2 of the *BEST1* gene (chr11:g.61951909G > A; c.103G > A; p.Glu35Lys) detected in heterozygous condition in the proband and the unaffected mother. **B** The variant in exon 4 of the *BEST1* gene (chr11:g.61955783C > A; c.313C > A), was detected in the proband but not in the unaffected mother. The reference sequence NM_004183 of *BEST1* was used

A heterozygous missense mutation, NM_004183.4(*BEST1*):c.103G > A, was found in exon 2 of the *BEST1* gene in both patients (chr11: g.61951909G > A; c.103G > A) and was further validated by Sanger sequencing (Fig. 4A). It resulted in the amino acid substitution of Glutamic acid (negatively charged) for Lysine (positively charged) at codon 35, NM_004183.4:p.(Glu35Lys) (Table 2). The variant is classified as likely pathogenic in the ClinVar database [31] and lies in the RFP-TM, chloride channel domain of the bestrophin protein. The NM_004183.4:p.(Glu35Lys) variant has not been reported in the 1000 genomes [36] and gnomAD databases [37] (accessed 30^th^ January 2022). The in-silico predictions according to PolyPhen-2 is to be probably damaging; and deleterious according to SIFT and MutationTaster2. The reference codon is evolutionarily conserved in mammals.

**Table 2:** Whole Exome Sequencing results of the *BEST1* gene of patients A and B

Gene (Transcript)	Location	Nucleotide change	Amino Acid change	Inheritance	PolyPhen-2 prediction	ClinVar Classification
*BEST1* (NM_004183.4)	Exon 2	c.103G > A	p.(Glu35Lys)	Autosomal recessive	Probably damaging	Uncertain significance
	Exon 4	c.313C > A	p.(Arg105Ser) **(novel)**	Autosomal recessive	Probably damaging	Uncertain significance

Another heterozygous missense mutation, NM_004183.4(*BEST1*):c.313C > A, was found in exon 4 of the *BEST1* gene in both patients (chr11:g.61955783C > A; c.313C > A), and was further validated by Sanger sequencing (Fig. 4B). It resulted in the amino acid substitution of Arginine (positively charged) for Serine (uncharged) at codon 105, NM_004183.4:p.(Arg105Ser) (Table 2). The variant lies in the RFP-TM, chloride channel domain of the bestrophin protein. The NM_004183.4:p.(Arg105Ser) variant has not been reported in the 1000 genomes databases [36] and has a minor allele frequency of 0.0007% in the gnomAD database (accessed 30^th^ January 2022) [37]. The in-silico predictions of the variant according to PolyPhen-2 is to be probably damaging, and deleterious according to SIFT and MutationTaster2. The reference codon is evolutionarily conserved in mammals. This mutation has not been previously reported in patients with ARB or VMD. The results of next-gen sequencing are summarized in Table 2*.* The IGV depicting the distribution of alternate allele and wild type allele for both variations is shown in Supplementary figure S 1A and S 1B*.*

We analyzed the mother for both variants through Sanger sequencing to clarify whether the two mutations were located on separate *BEST1* alleles. The NM_004183.4:p.(Glu35Lys) was detected in a heterozygous condition along with the wild type in the unaffected mother of the proband (Fig. 4A, B).

## Discussion

This report analyzed the clinical and imaging characteristics along with the genetic test results of two siblings with ARB. The diagnosis of ARB was established based on clinical observation and multimodal retinal imaging and further confirmed by whole exome sequencing and Sanger sequencing. Patient A demonstrated good central acuity, as seen in other patients with ARB in the first and second decade of life [42]. We noted that the sibling (patient B) had poor visual acuity in one eye due to amblyopia resulting from uncorrected esotropia. In addition, both the patients had short axial lengths in both eye without any abnormal iridocorneal anatomic features or shallow anterior chamber depth. Reduced axial length predisposes patients to angle-closure glaucoma, potentially leading to a further visual decline [42].

The whole exome sequencing revealed a likely compound heterozygous mutations in the *BEST1* gene shared by both siblings that likely led to ARB. The variants were validated by Sanger sequencing. One of the alleles carried a missense mutation in exon 2 NM_004183.4(*BEST1*):c.103G > A, which resulted in the amino acid substitution from negatively-charged Glutamic acid to positively-charged Lysine at the 35^th^ amino acid residue, NM_004183.4:p.(Glu35Lys). This variant was detected in the unaffected mother in a heterozygous state along with the wild type using Sanger sequencing. The variant has been submitted to ClinVar (accession number- RCV000356527) and has previously been reported by Tian et al. and Habibi et al., albeit in a homozygous state [43, 44]. To our knowledge, ours is the first study to report this variant in a compound heterozygous state.

Another variation was observed to be a transversion in exon 4 NM_004183.4(*BEST1*):c.313C > A, which resulted in the amino acid substitution from positively-charged Arginine to uncharged Serine at 105^th^ amino acid residue, NM_004183.4:p.(Arg105Ser) (Table 2). To our knowledge, this mutation has not been reported previously in patients with either ARB or VMD. This variant is predicted to be pathogenic. The NM_004183.4(*BEST1*):c.313C > A (p.Arg105Ser) has been submitted to ClinVar previously (accession number- RCV002025371.1) [31]. Two other disease-causing mutations affecting the same codon, p.Arg105Gly in patients with BMD, and p.Arg105Cys in a 69-year-old patient with age-related macular degeneration, have been reported [45, 46]. Interestingly, the p.Arg105Gly mutation resulted in additional extramacular multifocal deposits similar to ARB in three patients with BVMD [45].

**Fig. 5:**
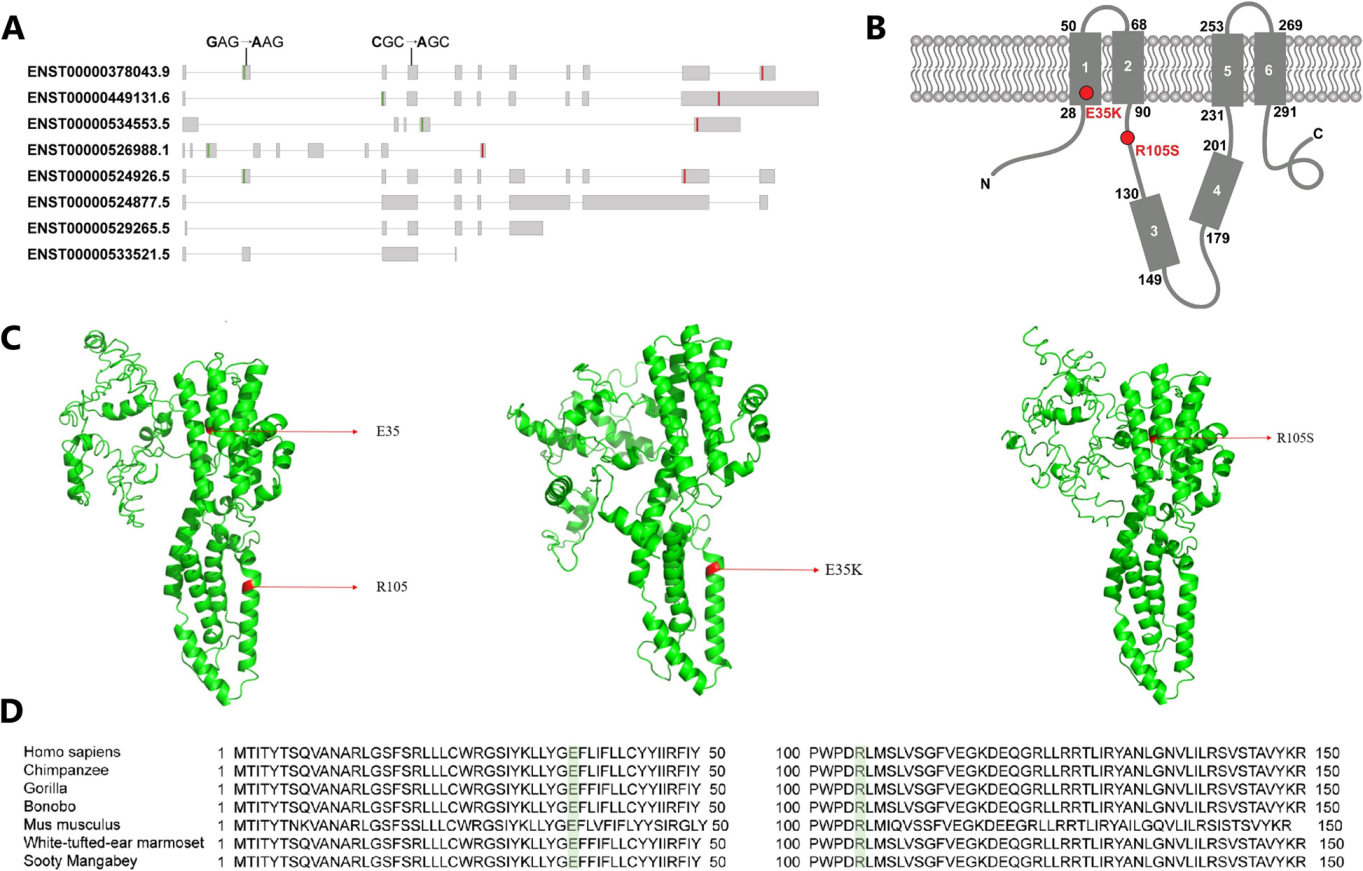
**A** Schematic representation of *BEST1* (NM_004183.4) transcript. Grey boxes represent exons, and lines connecting them represent introns. Green boxes represent the translation start site, while red boxes represent the translation termination site. **B** Topological representation of *BEST1* (Milenkovic et al.) representing mutation sites denoted by red circles. **C** Generated structural model of *BEST1* wild type, E35K, and R105S. **D** Multiple sequence alignment of *BEST1* from different species

Notably, the mutations discovered in this study are localized to the N-terminal region (Fig. 5A-C). The mutation NM_004183.4:p.(Glu35Lys) localizes to the first transmembrane domain, while the NM_004183.4:p.(Arg105Ser) mutation alters an amino acid in the cytoplasmic region distal to the second transmembrane domain (Fig. 5B). The amino acids at these positions are conserved among mammals (Fig. 5D). Among the roughly 335 mutations reported in *BEST1* thus far, only about 40 compound heterozygous and homozygous mutations are associated with ARB. [26, 34]

Although the detailed pathophysiology that leads to the disease is poorly understood, most of the characterized *BEST1* mutations alter the electrophysiological properties of the calcium-activated chloride channel (CaCC), which is thought to be determined by the N-terminus portion of *BEST1*, affecting the flow to chloride across the RPE [1, 5]. Crystallographic studies of the wild type and mutated proteins suggest that *BEST1* variants alter the cytoplasmic pore structure, which affects the permeability of anions or anion-cation selectivity, leading to lipofuscin accumulation and degeneration of the RPE (Fig. 5C) [47].

### What this study adds

This study expands the genetic spectrum of the *BEST1* variants associated with an ARB phenotype by reporting a novel variant p.(Arg105Ser), found in compound heterozygosity with another clinically significant variant in two affected siblings. Furthermore, the reported variant p.(Arg105Ser) variant appears to contribute to the ARB phenotype as the other variant alone did not cause any disease in the carrier (unaffected mother).

### Limitations

Although it does not affect the diagnosis, the genetic testing of the unaffected father and other unaffected siblings would have been ideal but could not be carried out.

## Supplementary Information

**Additional file 1.**

**Additional file 2.**

## Data Availability

All data generated or analyzed during this study are included in this published article. Accession number for the variants: SRR20990872.

## Abbreviations

AC: Anterior Chamber
ARB: Autosomal recessive bestrophinopathy
BCVA: Best-corrected visual acuity
BMD/BVMD: Best macular dystrophy/Best vitelliform macular dystrophy
EOG: Electroretinogram
ERG: Electrooculogram
FA: Fluorescein angiography
FAF: Fundus autofluorescence
GWAS: Genome-wide association studies
HGMD: Human Gene Mutation Database
NGS: Next-gen sequencing
OCT: Optical coherence tomography
OMIM: Online Mendelian Inheritance in Man
RPE: Retinal pigment epithelium
SIFT: Sorting Intolerant From Tolerant
SRF: Subretinal fluid
VMD: Vitelliform macular dystrophy
VUS: Variant of uncertain significance
