# Supplementary figures and images for: A novel compound heterozygous BEST1 gene mutation in two siblings causing autosomal recessive bestrophinopathy
Source: BMC Ophthalmol. 2022 Dec 16;22:493. doi: 10.1186/s12886-022-02703-5 (PMC9756692; doi:10.1186/s12886-022-02703-5)

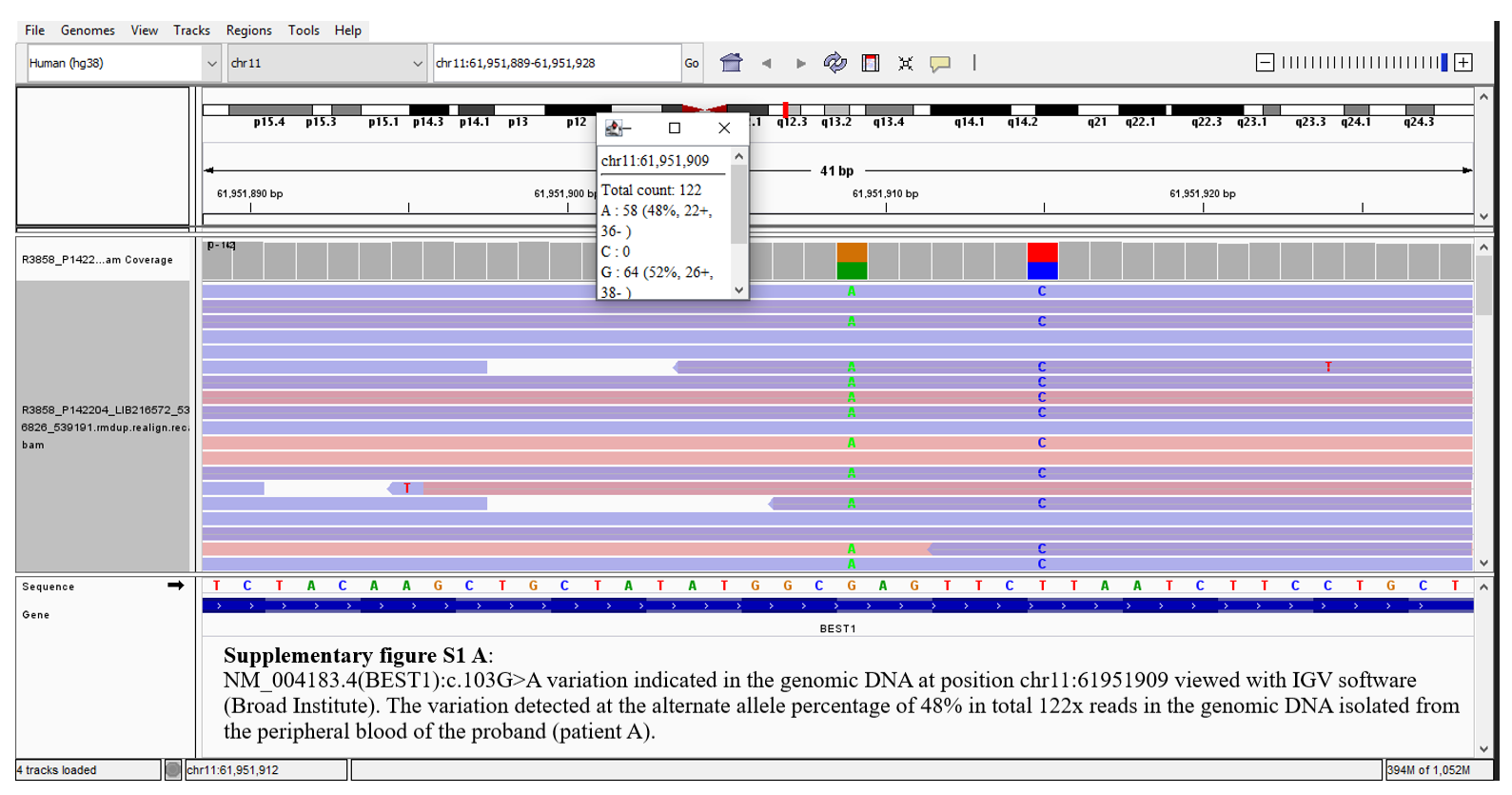

Supplement: Supplementary file 1 — Additional file 1. [file 12886_2022_2703_MOESM1_ESM.png]

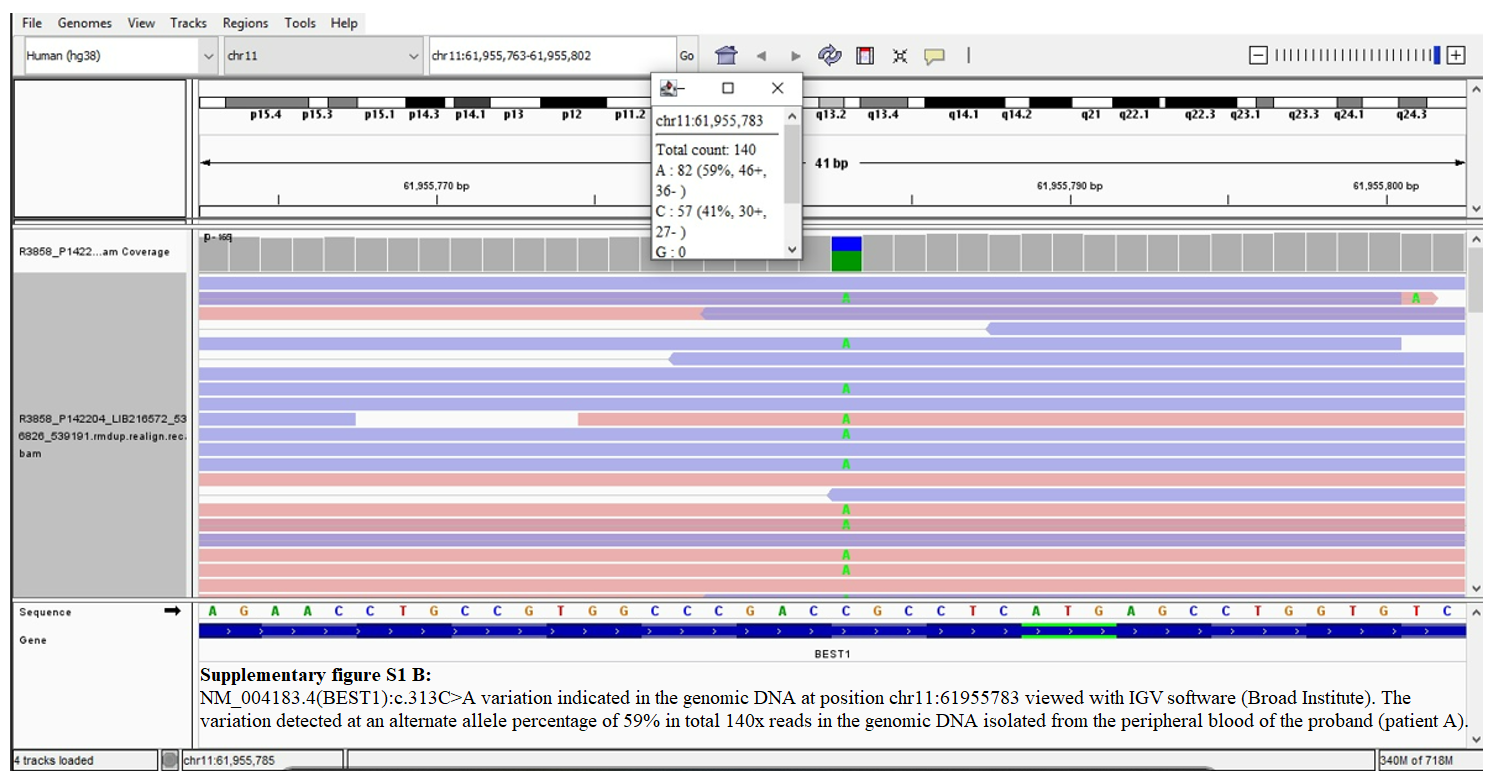

Supplement: Supplementary file 2 — Additional file 2. [file 12886_2022_2703_MOESM2_ESM.png]
